# Study protocol for the Anesthesiology Control Tower—Feedback Alerts to Supplement Treatments (ACTFAST-3) trial: a pilot randomized controlled trial in intraoperative telemedicine

**DOI:** 10.12688/f1000research.14897.2

**Published:** 2018-08-24

**Authors:** Stephen Gregory, Teresa M. Murray-Torres, Bradley A. Fritz, Arbi Ben Abdallah, Daniel L. Helsten, Troy S. Wildes, Anshuman Sharma, Michael S. Avidan, ACTFAST Study Group

**Affiliations:** 1 Department of Anesthesiology, Washington University School of Medicine, St. Louis, Missouri, 63110, USA

**Keywords:** telemedicine, decision support, protocol, randomized controlled trial

## Abstract

**Background**: Each year, over 300 million people undergo surgical procedures worldwide. Despite efforts to improve outcomes, postoperative morbidity and mortality are common. Many patients experience complications as a result of either medical error or failure to adhere to established clinical practice guidelines. This protocol describes a clinical trial comparing a telemedicine-based decision support system, the Anesthesiology Control Tower (ACT), with enhanced standard intraoperative care.

**Methods**: This study is a pragmatic, comparative effectiveness trial that will randomize approximately 12,000 adult surgical patients on an operating room (OR) level to a control or to an intervention group. All OR clinicians will have access to decision support software within the OR as a part of enhanced standard intraoperative care. The ACT will monitor patients in both groups and will provide additional support to the clinicians assigned to intervention ORs. Primary outcomes include blood glucose management and temperature management. Secondary outcomes will include surrogate, clinical, and economic outcomes, such as incidence of intraoperative hypotension, postoperative respiratory compromise, acute kidney injury, delirium, and volatile anesthetic utilization.

**Ethics and dissemination**: The ACTFAST-3 study has been approved by the Human Resource Protection Office (HRPO) at Washington University in St. Louis and is registered at clinicaltrials.gov ( NCT02830126). Recruitment for this protocol began in April 2017 and will end in December 2018. Dissemination of the findings of this study will occur via presentations at academic conferences, journal publications, and educational materials.

## Introduction

Each year, over 300 million surgical procedures are performed worldwide ^1^. Unfortunately, many patients will experience significant morbidity or mortality in the postoperative period ^2^. Research conducted at our institution and others has demonstrated an early postoperative mortality rate ranging from 1–5% and 90-day to 1-year mortality rates between 5–10% ^2– 13^. Additionally, 5–40% of patients will experience some type of postoperative surgical complication, including surgical site infection, respiratory complications, myocardial infarction, stroke and acute kidney injury, resulting in a three- to seven-fold increase in postoperative mortality ^3, 4, 11, 12, 14^.

Despite the overall decline in surgical morbidity and mortality over time, the risk of perioperative adverse events remains substantial ^2^. Some of this risk is a manifestation of either underlying patient pathology or the complexity of the surgical procedure itself, with increasingly complex registries and risk score calculators available to provide assessment of perioperative risk ^9, 12, 15, 16^. However, evidence also suggests that medical errors contribute considerably to negative patient outcomes ^17, 18^. Although some errors may be considered active, such as the administration of an incorrect medication, the failure to follow established clinical practice guidelines and recommendations likely has a more significant overall detrimental effect on patient outcomes. Prior studies have documented that deviation from evidence-based standards of care is commonin a variety of settings. This, deviation appears to worsen patient outcomes, including increases in surgical site infection, postoperative pneumonia, and mortality ^19– 25^.

Interventions to improve patient safety and outcomes remain a major focus in anesthesiology. The complexity of anesthetic practice can lead to frequent cognitive errors in the perioperative arena ^26, 27^, suggesting that the development of a real-time, tailored feedback system to support intraoperative decision-making may be valuable. The development of automated feedback and alerting systems has been demonstrated to improve adherence to a number of treatment guidelines ^28– 45^. However, the impact of decision support systems appears to decay over time ^46– 49^, and improvements in process variables may not translate into improved patient outcomes ^50^.

In the intensive care unit (ICU), the use of remote monitoring to augment care, commonly referred to as “telemedicine,” decreases ICU mortality and the length of ICU stay, and improves adherence to clinical practice guidelines ^51– 55^. While this type of clinical decision support has seen robust adoption in the critical care setting, its utilization in the intraoperative care of surgical patients is limited ^53^. In light of the benefits that have been demonstrated from using telemedicine in the ICU setting, we believe that the implementation of such a system in the operating room has the potential to elevate the general safety and quality of perioperative care.

We have designed a multifaceted approach for the development and institution of an Anesthesiology Control Tower (ACT) to provide real-time intraoperative telemedicine decision support. In the first component of our approach, we outlined a strategy of iterative usability testing and platform modification that allowed us to develop a high-fidelity, user-centered system ^56^. We intend to continue separate usability analyzes over the course of the pilot trial in order to evaluate the key usability elements of effectiveness, efficiency, and satisfaction ^57^ in a more real-world setting. Because the impact of a clinical intervention is dependent on the success of the process through which it is implemented ^58^, we will also evaluate implementation outcomes that are relevant to the use of the ACT in the perioperative setting ^59, 60^. In the second component of our approach, we will employ large-scale data analytics, integrating perioperative information in order to create forecasting algorithms for negative patient trajectories ^61^. In the current manuscript, we describe the third element of our investigation: a pilot randomized controlled trial that aims to demonstrate the superiority of the ACT in improving adherence to best care practices when compared to enhanced usual care.

## Methods and analysis

### Overview of research design

**Figure 1.**
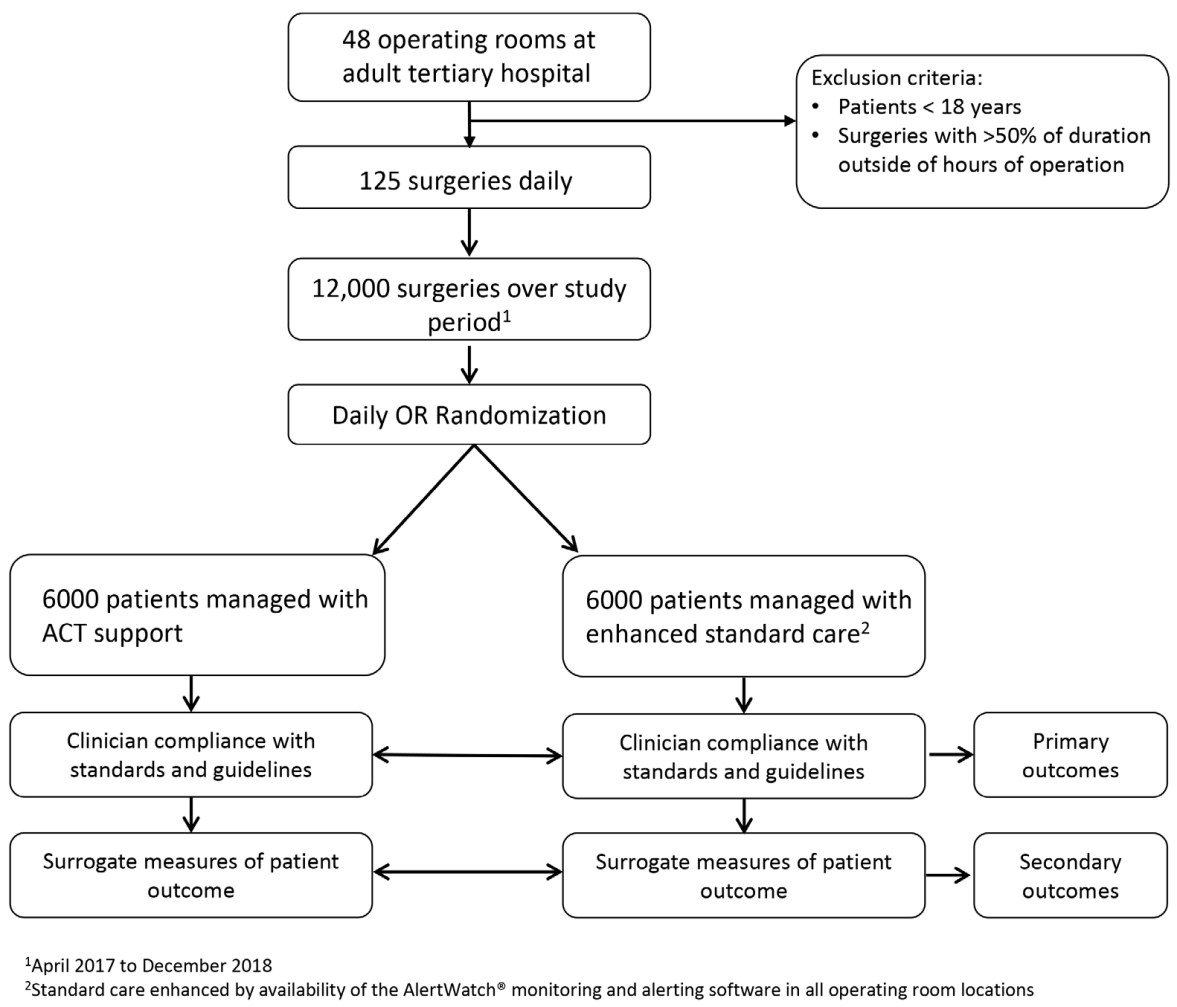
Flow diagram of study population.

The ACTFAST-3 study is a pragmatic comparative effectiveness trial that is taking place at an academic university-affiliated and adult tertiary care hospital in the United States that performs over 19,000 surgeries a year. We plan to enroll approximately 12,000 patients over the study period, with approximately 6,000 patients in the control arm and 6,000 patients in the intervention arm ( Figure 1). Patients will be included with a waiver of informed consent, as approved by the Human Research Protection Office (protocol number 201603038), as the risk associated with the ACT has been deemed to be minimal. Randomization will occur at the level of individual operating rooms on a daily basis.

**Figure 2.**
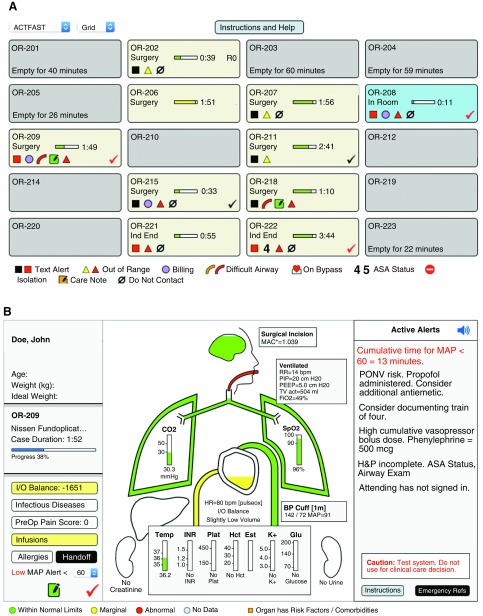
Interface of the AlertWatch ^®^ Control Tower system. ( **A**) AlertWatch ^®^ Control Tower Census View. This view shows summary information for operating rooms with ongoing procedures. Physiological alerts (e.g., low blood pressure) are shown as black or red squares, depending on the severity of the derangement, with red indicating a more severe abnormality. Checkmarks appear inside an operating room when an alert is triggered that has been classified as actionable and requires a response on the part of the clinicians in the Control Tower (see Figure 3). Control rooms are indicated with a “Do Not Contact” symbol. ( **B**) AlertWatch ^®^ Control Tower Patient Display View. This deidentified intraoperative patient display demonstrates organ-specific information individualized to each patient. Colors outlining organs indicate normal (green), marginal (yellow) or abnormal function (red). Orange would indicate an organ system at risk due to pre-existing conditions. The left side of the display shows patient characteristics and the case information. Lab values, if available, are listed beneath the kidneys. Alerts generated by the AlertWatch ^**®**^ system are listed on the right-hand side of the display. Specific alerts, determined by the study team to be clinically significant and actionable, trigger a checkmark to appear at the bottom left of the screen. This informs the Anesthesiology Control Tower (ACT) clinician that an alert is present that must be addressed. Clicking on this checkmark allows clinicians in the ACT to review and address these alerts ( Figure 3).

**Figure 3.**
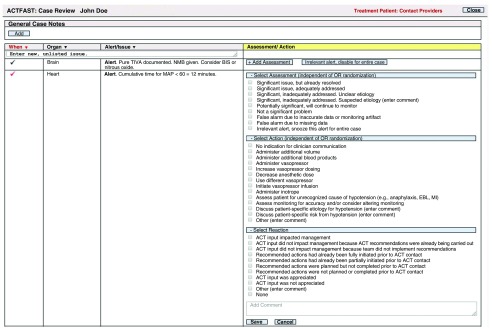
AlertWatch ^®^ Control Tower Case Review dialogue. Clinicians in the Anesthesiology Control Tower (ACT) use the Case Review window to address actionable Control Tower alerts, indicated by checkmarks on the Census View and the Patient Display. Within this Case Review window, clinicians document their assessment of the significant of each alert, what action they would recommend, and, in the case of intervention operating rooms (ORs), the reaction of the clinician in the OR to the ACT support.

The ACT will monitor all patients in both the control and intervention operating rooms using information gathered from the electronic medical record (EMR) and from a customized version of a perioperative monitoring and alerting program called AlertWatch® (Ann Arbor, MI). AlertWatch is an FDA-cleared (KI3O4OI) system that displays integrated patient information and alerts clinicians to physiologic derangements. It was recently demonstrated that use of the AlertWatch software was associated with improvements in several process measures, although this did not translate into an effect on clinical outcomes ^50^. For the purposes of our intervention, the commercially available AlertWatch platform was heavily modified through usability testing ^56^ to create a customized AlertWatch “Control Tower” mode that is only available within the ACT ( Figure 2 and Figure 3). The standard platform will remain available to all OR clinicians during this study. The ACT will provide clinicians in the intervention ORs with real-time feedback based on the available electronic resources, including AlertWatch Control Tower. Anesthesia providers in rooms assigned to the control group will also be monitored but will not receive decision support. Notably, the standard medical staffing models for providing an anesthetic will not be affected with this intervention, as the ACT is designed to augment decision-making, rather than replace critical team members.

The primary outcome measures in the ACTFAST-3 pilot study are compliance with best care practices for intraoperative core temperature management and intraoperative blood glucose management ( Table 1). These outcomes were selected because they are routinely and reliably tracked in the electronic medical record and optimal perioperative management of temperature and blood glucose is known to influence clinical outcome. We will also explore additional intraoperative process measures in addition to surrogate outcomes ( Table 2). The incidence of intraoperative hypotension and the incidence of postoperative renal dysfunction, atrial fibrillation, respiratory failure and delirium will be assessed via review of the EMR. Other postoperative complications, including intraoperative awareness, surgical site infection, readmission, and death will be assessed via analysis of the existing Center for Clinical Excellence Registry, American College of Surgeons’ National Surgical Quality Improvement Program (NSQIP) database, Society of Thoracic Surgery (STS) database, and Systematic Assessment and Targeted Improvement of Services Following Yearlong Surgical Outcomes Surveys (SATISFY-SOS) database ^62^. Outcomes related to the usability of the ACT intervention, including efficiency and efficacy of the software platform, will be obtained from AlertWatch data logs. These logs will also be used to obtain data related to the feasibility of implementing the pilot ACT. User satisfaction will be assessed through surveys administered to members of the anesthesia department.

**Table 1.** Primary outcome measures and definitions.

Measure	Outcome
Intraoperative temperature management	Proportion of patients with final recorded intraoperative core temperature greater than 36°C
Intraoperative blood glucose control	Proportion of cases with blood glucose ≥180 mg/dl upon arrival to the post-anesthesia recovery area

**Table 2.** Secondary outcome measures and definitions.

Intraoperative process measures	Outcomes
Intraoperative blood pressure management	Mean duration of time spent with Mean Arterial Pressure <60 mmHg
Temperature monitoring	Proportion of procedures lasting greater than 1 hour with documented temperature
Antibiotic dosing	Proportion of procedures with appropriate administration of repeat doses of antibiotics
Intraoperative blood glucose management	Proportion of cases with at least one dose of insulin administered for blood glucose greater than 180 mg/dl Intraoperative measurement of blood glucose in patients with type 1 diabetes undergoing cases ≥1 hour in length and patients with type 2 diabetes undergoing cases ≥2 hours in length
Train of four documentation	Proportion of cases with a train of four documented prior to extubation if a nondepolarizing neuromuscular blocking agent was administered
Ventilator management	Proportion of cases with median tidal volume less than 10 ml/kg ideal body mass
Volatile anesthetic utilization	Mean and standard deviation of fresh gas flow rates for cases with volatile anesthetic use >80% of case duration
Postoperative surrogate measures	Outcomes
Postoperative acute kidney injury	Incidence of individual outcomes ( Supplementary File 1)
Postoperative atrial fibrillation	
Postoperative respiratory failure	
Postoperative delirium	
Intraoperative awareness	
Surgical site infection	
30-day readmission	
30-day mortality	

### Study population, randomization, and blinding

The trial will include all adult patients undergoing surgery at two campuses of an academic university-associated hospital, Barnes-Jewish Hospital (South Campus and Parkview Tower) (St. Louis, MI, USA), between 7:00 AM and 4:00 PM Monday through Friday ( Figure 1). This includes a total of 48 operating room locations. The ACT will function on days when at least two anesthesia providers are available, one of whom must be an attending anesthesiologist. Patients undergoing surgical procedures with greater than 50% of the case length occurring outside of the ACT hours will be excluded from analysis. All patients younger than 18 will also be excluded from the study. Patients who undergo multiple surgeries in a single hospitalization or who have a second surgical procedure within 30 days of their initial surgery will be analyzed according to their initial randomization assignment. Patients returning for a second surgery more than 30 days after their initial surgical encounter will be considered as separate patients in the analysis. We will also obtain data from a group of historical control patients for the 6 months prior to the initiation of the ACTFAST-3 study, as part of an analysis related to potential sources of bias and contamination.

A randomization algorithm integrated into the AlertWatch system will direct patient group allocation on a daily basis. Due to the nature of the intervention in this study, clinicians working in the ACT and those randomized to receive support cannot be blinded to the intervention. To minimize any risk of bias with variation in ACT staff availability, we have ensured that OR-level randomization will performed each day in a 1:1 ratio. Researchers responsible for extracting data during the course of the study will be blinded to group allocation at the time of extraction.

### Primary intervention: ACT monitoring and decision support

A multidisciplinary team of clinicians in the ACT will remotely monitor all active operating rooms at the campus of interest. ACT clinicians will include attending anesthesiologists, anesthesiology fellows, anesthesiology residents, and certified and student registered nurse anesthetists. Information will be obtained in near real-time from multiple complementary sources, including the AlertWatch Control Tower software ( Figure 2) and the EMR. The clinicians in the ACT will use this information to communicate with OR clinicians to help maintain compliance with intraoperative best care practices and to assist with the detection and management of physiological derangements ^35, 63– 66^. These clinicians will evaluate all alerts generated by the AlertWatch Control Tower notification system ( Figure 3), including alerts from both the intervention and the control operating rooms. For ORs allocated to the intervention arm, the ACT will deliver decision support to the primary personnel caring for the patient via text message or telephone call. The clinician receiving the alert will determine the applicability of the alert to the clinical situation and will choose whether to carry out any recommendations sent by the ACT. In patients with a persistent critical event, the ACT will offer real-time assistance with crisis resource management.

 Operating rooms assigned to the control group will undergo the same monitoring and assessment by the ACT, but clinicians in these ORs will not receive any contact from the ACT. However, if clinicians staffing the ACT feel ethically obliged to contact a room assigned to the control group due to perceived potential for imminent and significant patient harm, they will be able to do so. Although we anticipate that this will be a rare occurrence, it will still be documented and reported as part of our study outcomes.

### Data collection and outcome measures

Data collection for this study will utilize multiple sources to extract outcome measures ^67^. All alert data generated by the AlertWatch Control Tower platform will be automatically logged to a secure database, including all responses by the providers in the ACT to individual alerts ( Figure 3). Data from the perioperative period will be imported from Metavision® (iMDsoft, Wakefield, Massachusetts, USA), the anesthesiology information management software system currently in use by the Department of Anesthesiology. In addition to capturing comprehensive intraoperative clinical data, Metavision® also stores preoperative information, such as patient characteristics, clinical and surgical history, comorbidities, and data from the immediate post-operative period. Of note, during the anticipated duration of this trial, our hospital system will be transitioning to Epic Systems software (Verona, WI, USA) for both the hospital electronic health record and the anesthesiology information management software. Postoperative data for patient outcomes will be obtained from the inpatient EMR record system, and from clinical registries ( SATISFY-SOS, NSQIP, STS).

### Primary outcome measures

The primary outcome measures in the ACTFAST-3 study are compliance with recommendations for intraoperative core temperature management and intraoperative blood glucose management ( Table 1). Data on primary outcomes measures will be recorded to an SQL server.

### Secondary outcome measures

Secondary intraoperative outcomes will include several process, surrogate, clinical measures ( Table 2). Intraoperative process outcomes will include blood pressure management, compliance with recommendations for repeat dosing of antibiotics and for temperature monitoring, management of hyperglycemia, documentation of train of four monitoring following neuromuscular blockade, and adherence to strategies for intraoperative low tidal volume ventilation. Additionally, the impact of the ACT on volatile anesthetic usage will be assessed. We will also evaluate surrogate and clinical outcomes, specifically, the incidence of postoperative acute kidney injury, postoperative atrial fibrillation, postoperative respiratory failure, postoperative delirium, intraoperative awareness, surgical site infection, 30-day hospital readmission, and 30-day mortality. Data will be obtained from review of electronic health records and cross-referencing of patients in the ACTFAST study with other surgical databases, as described above. We will also track the incidence of provider-reported intraoperative adverse events via a review of the departmental quality improvement database. Feasibility of implementing the ACT will be determined in part by examining the number of potentially staffed days versus the actual number of staffed days. Usability outcomes will include metrics such as the median number of alerts addressed by provider and across time.

### Data analysis

Comparisons between groups will be with parametric and non-parametric statistical tests, as appropriate. Fisher’s exact or χ ^2^ test will be used to evaluate primary outcome measures with regards to the following proportions: (i) the proportion of patients with a last-documented intraoperative core temperature greater than 36 degrees Celsius; and (ii) the proportion of patients arriving to the post-anesthesia care unit or ICU with a blood glucose greater than 180 mg/dl. Contingency statistical tests will be used to compare occurrence of hypothermia and hyperglycemia between groups. Secondary outcomes will be compared between groups using χ ^2^ or Fisher’s exact test for categorical outcomes, and two-sided t tests with unequal variances for comparison of means. By convention, statistical significance will be based on a two-sided p value <0.05. All statistical testing will performed using SAS® version 9.4 (SAS Institute Inc., Cary, North Carolina, USA). The small subset of rare patients in the control group whose provider may be contacted by the ACT clinicians out of concern for a significant patient safety event will be included in the control group in an intention-to-treat analysis. A sensitivity analysis will also be performed with inclusion of these patients in the intervention group. The frequency and rationale for contacting these rooms will be reported as part of our trial results.

Once the ACT intervention is executed, we anticipate several sources of contamination effect in the control group. There is a high likelihood of a robust Hawthorne effect due to OR clinician awareness of the ACT monitoring ^68, 69^. Also, all clinicians in the OR will eventually be included in the intervention group, due to the unit of randomization, and will likely become aware of the best management practices of interest in this trial. Therefore, even on days when they do not receive ACT support, clinicians may change their behavior, leading to overlapping improvements in both groups over the course of the study. Additionally, utilization of the AlertWatch software by clinicians in the ORs may increase over time. Learning effects might manifest most strongly among clinicians who staff the ACT and are therefore sensitized to the interventions and outcomes in this study. In order to evaluate the extent of the contamination and Hawthorne effects, we will collect baseline data for the group of historical controls. For categorical variables, contamination will be analyzed using logistic regression with a three-level categorical variable representing group assignment (historical cohort, control group, or intervention group); continuous variables will be analyzed using ANCOVA or non-parametric ANCOVA ^70^. Additionally, we will track which operating rooms utilize the AlertWatch system intraoperatively, and will plan to perform a subgroup analysis to assess the effect of the ACT in this subset of patients. We will also perform an analysis to ensure the integrity of the study data following our institutional transition to the Epic electronic medical record.

Within the AlertWatch system, all alerts that are generated are automatically logged to a secure database, as are all responses of the ACT clinicians to these alerts ( Figure 3). We will analyze these logs to determine how clinicians in the ACT monitor patients, address alerts, and interact with OR clinicians, and how OR clinicians respond to the ACT support. This data will allow us to explore aspects of the real-world usability of the ACT intervention related to efficiency and effectiveness, and will complement information gathered from qualitative usability surveys administered to department members.

### Sample size and power analysis

In this study, we plan to enroll a convenience sample of 12,000 patients over the course of the study period, based on the staffing available for the ACT and the usual daily surgical volume of approximately 125 cases. Power analysis was based on the two primary outcomes defined for this study, with the following assumptions:

i) Regarding the core-temperature outcome, we conservatively assumed that only 80% of Barnes-Jewish Hospital patients have their core temperature recorded during surgery. Among patients with their temperature documented, the target for this outcome was that the ACT intervention will increase the proportion of patients whose final recorded intraoperative temperature is above 36°C from 60% to 95%. For this calculation we assumed a standard deviation of core temperature of 0.9 degrees Celsius for both groups, based on an unpublished EMR audit.
ii) Regarding the primary outcome of glucose control, we assumed that the prevalence of diabetes mellitus among Barnes-Jewish Hospital surgical patients is about 20%, based on our EMR data over the past 5 years. Based on the same data, we also assumed that currently 60% of our diabetic patients reach a blood glucose >180 mg/dl at any point during surgery. Our goal was that the ACT intervention will reduce the proportion of patients arriving to the Post Anesthesia Care Unit (PACU) with a blood glucose value greater than 180 mg/dl from 60% to 40%.

A statistical power calculation based on the above assumptions was performed for each of the two primary study outcomes to determine whether the sample size (N=12,000) allocated for this study is adequate. The effective sample size for the study was defined as the largest sample needed to achieve any of the two stated outcomes. We mainly powered all targeted outcomes to detect a difference in proportions (adjusted for contamination between the two study groups) in a completely balanced clustered-randomized design study (24 operating rooms in each group) using two-sided Z-test statistics. We also assumed a minimum to 90% power, a significance level of 0.05, an intracluster correlation coefficient (ICC) varying between 0.01 and 0.05 by a small increment of 0.005, and a coefficient of variation of cluster sizes of 0.50. Table 3 shows the required sample per operating room as well as the overall sample needed to achieve the study targeted outcomes. The largest sample was required for the proportion of patients whose last recorded intraoperative core temperature is equal to or greater than 36°C (N=11,472). This value was sufficient for the other primary outcome.

**Table 3.** Sample size assumptions and calculations for primary outcomes.

Outcome †	Current practice	Cluster per group(size)		Target level *		Intracluster correlation coefficient	Total Sample Required
		Intervention	Control	Intervention	Control		
Core temperature: proportion reaching 36°C	50%	24(239)	24 (239)	95%	90%	0.0375	11,472
Post-operative Blood Glucose ≥180 mg/dL	60%	24(59)	24 (59)	40%	50%	0.03	2,832

†See Table 1 for full explanation of outcomes. *High contamination effects were set to reach 67% as 2 out of 3 physicians will participate in the ACT.

### Substudy in educational curriculum

While the primary goal of the ACTFAST-3 study is to evaluate the impact of the ACT on patient care and outcomes, the structure and environment of the ACT has allowed for the creation of a novel curriculum in perioperative medicine. The current educational paradigm for anesthesiology residents primarily focuses on the management of individual patients in the perioperative setting. However, the substantial increase in requirements for surgical procedures, a projected shortage of anesthesiologists, and financial constraints in healthcare suggest that it will eventually be infeasible for anesthesiologists to provide the level of supervision that is currently standard in the United States (e.g. one anesthesiologist for every one to four ORs) ^71^. There is currently little emphasis in anesthesiology education on process management and multitasking and caring for multiple patients in a complex care environment. With the support of the residency program director and departmental chair, we have revised the residency curriculum at our institute to allow each anesthesia resident to spend 2 weeks in the ACT during their final year of residency. We plan to implement an educational curriculum in perioperative telemedicine, focusing on the utilization of healthcare system resources to optimize intraoperative management, improve quality, and provide oversight of multiple patients undergoing complex surgical procedures.

### Adverse events and safety monitoring

We do not anticipate the occurrence of significant adverse events during this study. However, the primary investigator and the study team will review any adverse events identified by the departmental quality improvement program as potentially attributable to the ACT. The occurrence of any significant adverse events will be reported to the HRPO, and the study team and HRPO would decide together whether to halt the trial. No formal data-monitoring committee will used. There will be no audit of trial conduct during the investigation, although data recorded via the AlertWatch system will be reviewed and analyzed to determine appropriate group allocation and inclusion in the final analysis. No interim data analysis is planned for this pilot trial unless unanticipated safety issues are identified. There are no provisions for post-trial care or compensation to patients enrolled as part of this trial, as the intervention in the ACTFAST-3 trial involves only the addition of real-time decision-support tools and does not change existing anesthesia care models.

### Data management

The risk of breach of confidentiality will be minimized. The data necessary for the completion of the trial will be protected by passwords and is contained in applications that are compliant for protected healthcare information (PHI). AlertWatch meets this same standard of protection. Individual clinical alerts and the ACT evaluation of these alert will be documented using an electronic data capture tool in the AlertWatch system. Outcomes data will be stored on one of two Washington University Department of Anesthesiology servers (a SQL server or a Windows file server). Only trained employees of the Department of Anesthesiology or Barnes Jewish Healthcare are granted access to resources on this network. Access to the contents of this study will be further restricted to approved personnel only, using server-level permission access (for the SQL server), or Windows folder permission settings (for the file server). It is a strict policy that PHI cannot be saved or reviewed outside of this protected environment. Whenever possible, extracts for this project will avoid the use of this information. Data extracts can be reconnected to PHI using a special, non-PHI primary key, which this group has successfully used with previous studies.

### Strengths and limitations

The ACTFAST pilot study has important strengths. It is a randomized clinical trial conducted in a high volume, real world clinical setting and can be conducted efficiently, as many components of the proposed study are incorporated into existing infrastructures and processes at Washington University. This includes access to existing information technology resources and to established and ongoing registries (SATISFY-SOS, NSQIP and STS). The data required for analysis of the primary outcome measures are routinely recorded on every patient undergoing surgery at our institution, and the databases used for analysis of secondary surrogate and clinical outcomes also all have high levels of data fidelity.

Randomization of anesthesiology care teams can be easily implemented, and the process for providing feedback alerts does not require any advanced preparation on the part of clinicians working in the OR. These clinicians will participate in the ACTFAST trial in the course of their routine clinical work, and the impact on overall workflow and workload will be minimized through the testing in our first phase of the study ^56^. We anticipate that it will be feasible to staff the ACT during the pilot RCT. The feasibility is enhanced by participation of a highly committed cadre of attending anesthesiologists and all of the residents in the anesthesiology department, as well as an experienced team of investigators that has established a track record of collaboration and completion of major clinical trials.

 The following limitations should be considered. The AlertWatch software is currently available on all computers in the OR, and in-room provider utilization of AlertWatch may increase over the course of the study. In response, we plan to conduct a subgroup analysis with user log-in data to ascertain the impact of in-room software utilization, defined as documentation of intraoperative provider log-in to the AlertWatch system. Also, the ACTFAST study will be vulnerable both to Hawthorne and contamination effects. While we do not think that these effects can be eliminated, we have considered how best to account for them in the analyses. An important constraint and possible source of bias will be that it will not be possible to ensure blinding of OR clinicians as any communication from the ACT will inform them that their operating room is in the intervention group on that day ^72^. However, clinicians outside of the OR, and the researchers responsible for extracting data, will be blinded to group assignment.

Another potential source of bias involves the existing surgical databases that will be used during analysis (i.e. STS, NSQIP, SATISFY-SOS). These registries themselves may be biased according to which patients choose to participate, with individual patients’ outcomes impacting their willingness or ability to provide reliable information, and which patients are contactable. We have been attempting to mitigate this source of bias by employing three modalities (e-mail, telephone and mail) to reach patients postoperatively in one such study ^62^. Overall, the registries have impressive response rates, and there does not appear to be systematic bias in any of these registries based on baseline patient characteristics. Therefore, we expect our data sources to be robust, with minimal deficiencies.

### Ethics and dissemination

This study was approved by the HRPO at Washington University (St. Louis, MI, USA, protocol number 201603038). This protocol is written in compliance with the Standard Protocol Items: Recommendations for Interventional Trials (SPIRIT) checklist with consideration of the Consolidated Standards of Reporting Trials (CONSORT) guidelines ^73, 74^.

If the results of the pilot ACTFAST-3 trial show benefit, the pilot study will likely be replicated as a larger, multicenter study for further validation that this intervention remains beneficial and that it is feasible to institute at other centers. We also anticipate the expansion of the ACT into the surrounding healthcare facilities within our hospital system. Larger trials could focus on expanded clinical and patient-reported outcomes (e.g. death, renal failure, delirium, duration of mechanical ventilation, intensive care length of stay, post-discharge disposition, postoperative falls, return to work, disability-free survival). The ACT infrastructure could also be used to explore current controversies in perioperative care by testing candidate experimental interventions (e.g., fluid management strategies, blood transfusion triggers). We envision that national implementation of the ACT concept would occur, which would be comparable to the path that similar programs for intensive care units have followed.

Any significant changes to the protocol or the analysis plan during the trial will be communicated directly to the Washington University HRPO, as well as via update of the ACTFAST-3 registration at clinicaltrials.gov (ClinicalTrials.gov Identifier: NCT02830126). We also plan to publish any modifications made to this protocol during dissemination of the results of the trial. Authorship for the final trial data will be determined in accordance with International Committee of Medical Journal Editors (ICMJE) guidelines.

### Data sharing

Data from the ACTFAST-3 trial will be made available for analysis in compliance with the recommendations of the ICMJE ^75^. For this study, individual participant data that underlie the results of the trial will be made available after appropriate deidentification, along with the study protocol and statistical analysis plan. We plan to make this information accessible to researchers who provide a methodologically appropriate proposal for the purpose of achieving the aims of that proposal. Data will be available beginning 9 months and ending 36 months following trial publication at a third-party website. Data requestors will need to sign a data access agreement to gain access to trial data. Proposals should be directed to avidanm@wustl.edu.

## Conclusions

Despite aggressive efforts aimed to improve the quality of perioperative care, the risk of morbidity and mortality following a major surgical procedure remains substantial. In this protocol, we describe a pilot pragmatic, randomized, controlled trial in intraoperative telemedicine that examines the ability of a novel system of real-time feedback to improve adherence to perioperative best care practices. We hypothesize that the implementation of the ACT will be feasible and that it will increase clinician compliance with clinical practice standards. The development of the ACT, as described in this protocol, will also lay the groundwork for a subsequent large randomized controlled trial examining the utility of the ACT in improving patient outcomes following surgical procedures.

The findings from the trial will be disseminated in the form of posters and oral presentations at scientific conferences, as well as publications in peer-reviewed journals. Updates and results of the study will be available at https://clinicaltrials.gov/ct2/show/NCT02830126.

## Data availability

No data is associated with this study.

## Supplementary material

**Supplementary File 1: Definitions of postoperative surrogate measures for the ACTFAST-3 clinical trial.**
