# Supplementary material for: Study protocol for the Anesthesiology Control Tower—Feedback Alerts to Supplement Treatments (ACTFAST-3) trial: a pilot randomized controlled trial in intraoperative telemedicine
Source: F1000Res. 2018 Aug 24;7:623. Originally published 2018 May 22. [Version 2] doi: 10.12688/f1000research.14897.2 (PMC6039946; doi:10.12688/f1000research.14897.2)
Supplement: Supplementary file 1 [file f1000research-7-17085-s0000.tgz › a27c0832-beec-48d2-beb5-bf8e639da154.docx]

Supplementary File 1: Definitions of postoperative surrogate measures for the ACTFAST-3 clinical trial.

| **Outcome** | **Definition** |
| --- | --- |
| Postoperative acute kidney injury | Diagnosed when any of the following three criteria are met: (i) an increase in serum creatinine by 50% compared with preoperative within 7 days, (ii) any increase in serum creatinine > 0.3 mg/dL in 48 hours, or (iii) oliguria (urine output <0.5 mL/kg/hr for 6-12 hours). |
| Postoperative atrial fibrillation | New-onset postoperative atrial fibrillation in a patient with no prior history of atrial fibrillation. |
| Postoperative respiratory failure | Mechanical ventilation for greater than 24 hours after surgery, or unplanned postoperative re-intubation and mechanical ventilation within 30 days of surgery. |
| Postoperative delirium | The presence of a positive CAM-ICU assessment in the postoperative period |
| Intraoperative awareness | Awareness will be elicited with a modified question from the Brice questionnaire, inquiring whether the patient remembers anything between going to sleep at the beginning of the surgery and waking up at the end of the surgery. The awareness experience will be classified based on the Michigan Awareness Classification Instrument. |
| Surgical site infection | Infection at the site of surgery (assessed as part of the SATISFY-SOS registry) |
| 30-day readmission | Readmission to the hospital within 30 days of initial surgery |
| 30-day mortality | Death of any cause occurring in or out of the hospital, within 30 days of the index surgery |
