# Supplementary material for: Study protocol for the Anesthesiology Control Tower—Feedback Alerts to Supplement Treatments (ACTFAST-3) trial: a pilot randomized controlled trial in intraoperative telemedicine
Source: F1000Res. 2018 Aug 24;7:623. Originally published 2018 May 22. [Version 2] doi: 10.12688/f1000research.14897.2 (PMC6039946; doi:10.12688/f1000research.14897.2)
Supplement: Peer review history

# Peer review: Referee response for version 1

**DOI:** 10.5256/f1000research.16217.r35259

**Published:** 2018-06-29

**Authors:** Morten H. Bestle, Christian Ari Dalby Sørensen

**Affiliations:** 1 Department of Anaesthesiology and Intensive Care, Nordsjællands Hospital - Hillerød, University of Copenhagen, Hillerød, Denmark

Thank you for the opportunity to review this paper.

 In manuscript the authors describe a pilot randomized controlled trial that aims to demonstrate the implementation and utility of the anesthesiology control tower (ACT) in improving adherence to best care practices when compared to enhanced usual care. The authors propose to randomize 12,000 patients over the study period, with approximately 6,000 patients in the control arm and 6,000 patients in the intervention arm. Clinicians grouped in the intervention arm will be provided with real-time feedback based on the available electronic resources. Primary and secondary outcomes will be compared to the control group.

    

 Page 3 paragraph 2: The authors state that some of the risks of perioperative adverse events may be a manifestation of either underlying patient pathology or the complexity of the surgical procedure itself. The authors could consider elaborating that statement in more details. How big is the proportion of underlying patient pathology and complex surgical procedures?

 Page 3 paragraph 2: The authors state that prior studies have documented that deviation from evidence-based standards of care is common, and that deviation results in poorer patient outcomes. Which outcomes has been the focus of prior studies?

 Page 3 paragraph 8: Why have you chosen these outcomes to be the primary outcomes?

 Page 4 paragraph 2: The authors mentions that only patients undergoing surgery between 7:00 AM and 4:00 PM Monday through Friday will be included. Have you considered there could be a difference between elective and acute surgery. Are clinicians more prone to follow clinical guidelines at day time compared to night time?

We have read this submission. We believe that we have an appropriate level of expertise to confirm that it is of an acceptable scientific standard.

# Response

**Published:** 2018-07-16

**Authors:** Teresa Murray-Torres

**Affiliations:** Washington University in Saint Louis, USA

Thank you for taking the time to review our manuscript and provide feedback. We have submitted a revised version of our protocol addressing the reviewer's comments. Our changes in response to the referee are as follows: 

 Page 3 paragraph 2: The authors state that some of the risks of perioperative adverse events may be a manifestation of either underlying patient pathology or the complexity of the surgical procedure itself. The authors could consider elaborating that statement in more details. How big is the proportion of underlying patient pathology and complex surgical procedures?

*We have expanded this sentence to highlight the development of complex surgical risk calculators to evaluate perioperative risk using both patient pathology and the surgical procedure.*

 Page 3 paragraph 2: The authors state that prior studies have documented that deviation from evidence-based standards of care is common, and that deviation results in poorer patient outcomes. Which outcomes has been the focus of prior studies?

*We have updated this section to highlight that deviation from evidence-based standards of care is ubiquitous across a variety of health care settings and may be associated with an increase in a number of adverse patient outcomes, including surgical site infection, pneumonia, and mortality.*

 Page 3 paragraph 8: Why have you chosen these outcomes to be the primary outcomes?

*These outcomes were selected because they are routinely and reliably tracked in the electronic medical record and optimal perioperative management of temperature and blood glucose is known to influence clinical outcome. We have added this information to the manuscript.*

 Page 4 paragraph 2: The authors mentions that only patients undergoing surgery between 7:00 AM and 4:00 PM Monday through Friday will be included. Have you considered there could be a difference between elective and acute surgery. Are clinicians more prone to follow clinical guidelines at day time compared to night time?

*We do recognize that this is a limitation of our current study, but we have attempted to account for any variation in guideline compliance during off-hours by equally applying time exclusion criteria to both our control and intervention ORs. Additionally, we have designated that patients having a surgical procedure with >50% of the operative time occurring outside of ACT hours will be excluded from analysis. Evaluating variations in compliance with perioperative guidelines outside of normal working hours is an interesting proposal, and may be considered as part of a future expansion of the ACT concept.*

# Peer review: Referee response for version 1

**DOI:** 10.5256/f1000research.16217.r34272

**Published:** 2018-06-05

**Authors:** Leif Saager, Michael Burns

**Affiliations:** 1 University of Michigan, Ann Arbor, MI, USA

Thank you very much for the opportunity to review this innovative and timely submission.

 The manuscript is eloquently written and the study protocol comprehensively described; our comments are therefore few and minor.

 In this article the authors present a study protocol for a randomized control trial in the field of intraoperative clinical decision support. The authors propose to randomize 12,000 patients to either intraoperative clinical decision support or enhanced intraoperative clinical decision support by utilizing a novel Anesthesia Control Tower (ACT) concept. Throughout the article the authors thoroughly present their pragmatic study with adequate details and a thoughtful patient-centric approach. Their identification of the complexity of the anesthetic practice and cognitive requirements is well founded, and their reference to the ICU remote monitoring systems is established.

   

 On page 3, paragraph 1, the authors state that “10-40% of patients will experience some sort of postoperative surgical complication”. The citations mostly refer to elderly and/or high-risk surgical patients. Perhaps the authors could consider adding a reference for a general surgical population.

 On page 4, the authors state the ACT will function only on days with at least 2 anesthesia providers available. Could this introduce bias into the study as on OR days with high volume, or complex cases requiring lower staffing ratios, the availability of staff for the ACT would be less likely?

 On page 7, paragraph 2, the authors state an anticipated transition in electronic health records. In our experience, implementation of a new record keeping system can increase cognitive load, documentation errors, and lags in data acquisition. Our concern would be a possible compromise of study data. Do the authors have a contingency/transition plan available?

 On page 8, the authors base the sample size calculation on core temperature measurements. The rest of the manuscript is less specific as to the site of temperature measurement. Will only core temperatures be utilized in this study?

 On page 9, paragraph 2, the authors propose an innovative educational curriculum. Would the authors consider providing more detail on the implementation and evaluation of this component?

 In Table 2, the authors describe secondary outcomes. Would it be possible to add an appendix to provide definitions for these parameters or reference NSQIP/STS documents as the source of these definitions?

We have read this submission. We believe that we have an appropriate level of expertise to confirm that it is of an acceptable scientific standard.

# Response

**Published:** 2018-07-16

**Authors:** Teresa Murray-Torres

**Affiliations:** Washington University in Saint Louis, USA

Thank you for taking the time to review our manuscript and provide feedback. We have submitted a revised version of our protocol addressing the reviewer's comments. Our changes in response to the referee are as follows: 

 On page 3, paragraph 1, the authors state that “10-40% of patients will experience some sort of postoperative surgical complication”. The citations mostly refer to elderly and/or high-risk surgical patients. Perhaps the authors could consider adding a reference for a general surgical population.

*We have updated this statistic to “5-40%,” including a reference examining NSQIP complication rates in patients undergoing orthopedic surgical procedures, primarily elective total joint procedures.*

 On page 4, the authors state the ACT will function only on days with at least 2 anesthesia providers available. Could this introduce bias into the study as on OR days with high volume, or complex cases requiring lower staffing ratios, the availability of staff for the ACT would be less likely?

*We have attempted to minimize the risk of bias secondary to ACT staff availability by performing OR randomization each day with a 1:1 allocation. We anticipate that this will allow for any staffing variations to equally affect both the intervention and control groups to minimize bias. We have updated the manuscript to specifically address this point.*

 On page 7, paragraph 2, the authors state an anticipated transition in electronic health records. In our experience, implementation of a new record keeping system can increase cognitive load, documentation errors, and lags in data acquisition. Our concern would be a possible compromise of study data. Do the authors have a contingency/transition plan available?

*Fortunately, the data required to evaluate the primary and secondary outcomes in this study is electronically populated from patient monitoring data (temperature) or autopopulated into the electronic medical record following measurement (glucose). Although we do not anticipate any significant difficulties with ensuring the integrity of the study data, we do plan to perform an analysis to confirm that there has been no significant compromise of study data.*

 On page 8, the authors base the sample size calculation on core temperature measurements. The rest of the manuscript is less specific as to the site of temperature measurement. Will only core temperatures be utilized in this study?

*Yes, we plan to only utilize core temperature in our analysis of temperature as a primary outcome. This has been updated in the manuscript.*

 On page 9, paragraph 2, the authors propose an innovative educational curriculum. Would the authors consider providing more detail on the implementation and evaluation of this component?

*At present time, we are still actively developing the educational curriculum for residents rotating through the ACT. The specific endpoints for the protocol assessing this substudy are not yet defined.*

 In Table 2, the authors describe secondary outcomes. Would it be possible to add an appendix to provide definitions for these parameters or reference NSQIP/STS documents as the source of these definitions?

*We have added an appendix to define the postoperative surrogate outcomes for the study.*
